# Determinants of partial and adequate maternal health services utilization in Nigeria: analysis of cross-sectional survey

**DOI:** 10.1186/s12884-023-05712-4

**Published:** 2023-06-20

**Authors:** Sulaimon T. Adedokun, Olalekan A. Uthman, Luqman A. Bisiriyu

**Affiliations:** 1 Department of Demography and Social Statistics, Obafemi Awolowo University, Ile-Ife, Nigeria; 2 Warwick-Centre for Applied Health Research and Delivery (WCAHRD), Division of Health Sciences, University of Warwick Medical School, Coventry, UK; 3 Centre for Evidence-based Health Care, Faculty of Medicine and Health Sciences, Stellenbosch University, Cape Town, South Africa

**Keywords:** Health Services, Maternal, Utilization, Multinomial, Antenatal, Partial, Adequate

## Abstract

**Background:**

Access to health services during pregnancy, childbirth and the period after birth provides a substantial opportunity to limit cases of maternal mortality. In sub-Saharan Africa, the proportions of women who utilize health services remain below 70%. This study examined the factors associated with partial and adequate maternal health services utilization in Nigeria.

**Methods:**

This paper used data from 2018 Nigeria Demographic and Health Survey (DHS) comprising 21,792 women aged 15–49 years who had given births within five years of the survey. The study focused on antenatal care attendance, place of birth and postnatal care using a combined model. Multinomial logistic regression was applied in the analysis.

**Results:**

About 74% of the women attended antenatal care, 41% gave birth in health facilities and 21% attended postnatal care. While 68% of the women partially utilized health services, 11% adequately utilized the services. The odds of partially and adequately utilizing health services increased for ever married women, women with secondary or higher education, from richest households, living in urban area, having no problem either getting permission to visit health facility or reaching health facility.

**Conclusions:**

This study has revealed the factors associated with partial and adequate utilization of maternal health services in Nigeria. Such factors include education, household wealth, marital status, employment status, residence, region, media exposure, getting permission to use health service, unwillingness to visit health facility without being accompanied and distance to health facility. Efforts aimed at improving maternal health services utilization should place emphasis on these factors.

**Supplementary Information:**

The online version contains supplementary material available at 10.1186/s12884-023-05712-4.

## Background

Although maternal mortality decreased globally by nearly 44% from 1990 to 2015 [1], maternal health is still a major concern in low resource settings. Available records show that 66% of global maternal mortality occurred in sub-Saharan Africa [1]. Most deaths are linked to complications arising during and following birth [2]. Access to health services during pregnancy, childbirth and the period after birth provides a substantial opportunity to limit cases of maternal mortality [2, 3]. It is in view of this that the World Health Organization (WHO) and other international bodies embarked on efforts that promote health services use and ensure quality care throughout the duration of pregnancy, childbirth and post-birth period. WHO introduced some recommendations on antenatal care in order to ensure that women have a positive pregnancy experience. These recommendations comprise: (i) nutritional interventions which focus on provision of adequate information on relevant diet; (ii) maternal and foetal assessment for early and proper diagnosis of ailment such as anaemia and asymptomatic bacteriuria (ASB); (iii) preventive measures which include tetanus toxoid vaccination and treatment to protect against malaria; and; (iv) health system interventions [4]. In another development, recommendations were promoted in respect of childbirth and post-delivery stage. Those involving childbirth include induction of labour, diagnosis of delay in the first stage of labour, prevention of delay in the first stage of labour, treatment when such delay occurs and care during labour augmentation [5]. For postnatal stage, attention is paid to timing of discharge from health facility, timing and number of postnatal contacts and content of postnatal care for the mother [5].

Records show that globally, 66% of women attended antenatal care, 80% had institutional delivery and 61% used postnatal care service [6–8]. While regional distributions for antenatal care attendance indicate 55% for South Asia, 88% for East Asia and the Pacific and 91% for Latin America and the Caribbean, distributions by institutional delivery indicate 82% for South Asia, 91% for East Asia and the Pacific and 94% for Latin America and the Caribbean [6, 7]. Meanwhile, proportions of women who utilize health services in sub-Saharan Africa remain below 70%. While 55% received at least four antenatal visits, 66% delivered in health facility and 53% utilized postnatal care [9–11]. In Nigeria, efforts have also been made to improve the health services utilization of women through the establishment of maternal and child health programme. This programme which took off in 2010, aimed at improving the use of routine services; strengthening the health system; increasing antenatal care uptake; improving health workers skills in delivering maternal, new-born and child interventions and; improving health information management systems utilization [12]. In spite of these efforts, evidence shows that 52% of women had at least four antenatal visits, 38% delivered at health facility and 37% utilized postnatal care [13–15]. Inadequate utilization of health services among women has been attributed to a host of factors such as age, education, distance to facility, poverty, household wealth, place of delivery, residence, media exposure, child’s birth order, transport facilities and economic factors [15–24].

Most of these studies concentrated their focus on single outcome of maternal health services utilization by considering only antenatal care, childbirth or postnatal care at a time. Extending the research beyond single outcome would provide a better insight into the factors that predict health services utilization which would enhance a robust explanation for such factors. This paper, therefore, examines the correlates of antenatal, delivery and postnatal care in a single analytical framework. The paper is also considering these three maternal health services because they constitute the major components in the new coverage targets and milestones that need to be achieved by 2025 if the Sustainable Development Goals (SDGs) are to be met [25].

## Methods

### Study design and sampling procedure

The data used in this study were obtained from 2018 Nigeria Demographic and Health Survey (NDHS), which makes it a secondary dataset. The survey is cross-sectional, nationally representative and designed to provide information on population and health at national, regional and state levels. It was conducted by the National Population Commission (NPC) with technical support provided by ICF. While the response rate for the survey was 99%, the country was divided into administrative units. These administrative units include states, local government areas and localities. Apart from these administrative units, enumeration areas were created from localities based on the last census exercise. The sample for the survey was selected using a stratified two-stage cluster design. While the first stage involved selection of 1400 enumeration areas, 30 households were selected in each enumeration area in the second stage. This resulted in the selection of 42,000 households. Information was obtained from 42,821 women aged 15–49 in the selected households using a standardized questionnaire that was administered through face-to-face interview. The questionnaire was designed to capture information on background characteristics, antenatal, delivery and postnatal care, child immunization and childhood illnesses, reproductive history and child mortality, among others. All women of reproductive age who were either permanent residents of the households or visitors present in the households on the night preceding the survey were eligible for interview. Ethical clearance was obtained by DHS Program from appropriate authorities and this has been stated in the declarations section of this study. Detailed description of sampling technique and data collection procedure used have been published elsewhere [26].

### Outcome variable

This study was limited to women aged 15–49 who gave births within five years of the survey. The outcome variable was derived from three variables: antenatal care visits, place of birth and postnatal care visits. Antenatal care visit was measured as a binary outcome having 0 for women who did not attend clinic at all and 1 for those who attended. Place of birth was defined as 0 for those who delivered outside health facilities and 1 for those who had facility births. Postnatal care was dichotomised as 0 for those who did not report for check-up within two months after birth and 1 for those who reported for check-up. These three variables were thereafter combined to arrive at a single outcome variable called maternal health services use with three categories namely: not utilized (for those who did not utilize any of the three services); partially utilized (for those who used one or two of the services) and; adequately utilized (for those who utilized all the services). This type of categorization of maternal health service utilization has been used in a previous study where the outcome variable was defined as no care, partial care and full care [27].

### Independent variables

The following variables constituted the independent variables in the study: age, education, marital status, employment status, household wealth, preceding birth interval, residence and region. Others include media exposure, getting permission to use health facilities, getting money for treatment, distance to health facilities and not willing to go to health facilities alone. Age was defined as 15–24 years, 25–34 years and 35 years and above. Education was measured as none, primary and secondary/higher. Marital status was divided into never and ever married. Employment status was grouped into working and not working. Household wealth was categorised into poorest, poorer, middle, richer and richest quintiles. Preceding birth interval was dichotomised as less than 24 months and 24 months or more. Residence was grouped into urban and rural. Region was divided into North Central, North East, North West, South East, South West and South South. Media exposure was defined as not exposed and exposed. Getting permission to use health facilities refers to the consent a woman seeks from her spouse before visiting. This was categorised as being a problem and not a problem. Getting money for treatment was grouped into being a problem and not a problem. The distance a woman travels before reaching a health facility was measured in terms of whether such a woman considered the distance a problem or not. Unwillingness to go to health facilities alone refers to a situation where a woman would need the company, most probably, of her spouse before visiting health facilities. Unwillingness was measured as being a problem and not a problem. All the responses with ‘a problem’ and ‘not a problem’ categories were obtained directly from the women.

### Statistical analyses

Descriptive statistics was used to present distributions of the independent variables against the outcome variable together with their $p$ values. We obtained the sample weight *(v005)* and calculated the weighting factor ($\frac{v005}{1,000,000}$) which was then applied to the data through *svyset* command which was used to control for over-reporting and under-reporting in the survey. Multinomial logistic regression model was used for the multivariable analysis. The use of multinomial model was necessitated by the categories of outcome variable which are more than two. The multinomial logistic regression model is given as:

$$\begin{array}{c}In\left( {\frac{{\pi \left( {Y = j|{x_1},{x_2}, \ldots ,{x_p}} \right)}}{{\pi \left( {Y = J|{x_1},{x_2}, \ldots ,{x_p}_1} \right)}}} \right) = {\alpha _j} + {\beta _{j1}}{X_1}\\+ {\beta _{j2}}{X_2} + \ldots + {\beta _{jp}}{X_p}\end{array}$$

Where $j=\text{1,2}, \dots , J-1$ (categories of the outcome variable) and $J$ is the base outcome; ${\alpha }_{j}$ represents the intercepts and ${\beta }_{j1}$, … ${\beta }_{jp}$ represent the logit coefficients; and ${X}_{1}\dots {X}_{p}$ represent the independent variables [28].

All variables that were found to be significant at descriptive analysis level were included in the final multinomial logistic models. The base outcome in the analysis is not utilized (those that never used any of the services). After obtaining the logit coefficients, we exponentiated to arrive at odds ratios (ORs) for easy interpretation. Three α levels (0.05, 0.01 and 0.001) were considered statistically significant in respect of the results obtained. Stata statistical software version 14 was used to perform all the analyses [29].

## Results

### Descriptive statistics

The results of the descriptive analysis are presented in Tables 1 and 2. The analysis included a total of 21,792 women who used or did not use health service during pregnancy, delivery or postnatal period. Results in Table 1 show that 74% of the women attended antenatal care, 41% delivered at health facility and 21% attended postnatal care. Women with secondary or higher education had the highest utilization rates for antenatal care (90%), facility delivery (69%) and postnatal care (30%). Utilization of health services among women in respect of household wealth indicates that those from richest households had the highest rates. Close to 92% of women in this category attended antenatal care, 80% delivered at health facility and 31% attended postnatal care. About 77% of those who reported that getting permission to visit health facility was not a problem attended antenatal care and 44% had facility delivery. While 80% of women who reported that they did not experience any problem in respect of the distance to health facility attended antenatal care, 46% and 22% respectively delivered at health facility and attended postnatal care. The same situation is observed among the women in respect of willingness to go to health facility alone. About 77% of those who claimed that they did not have any problem going to health facility alone attended antenatal care, 44% delivered at health facility and 21% attended postnatal care. Results from Table 2 reveal that 68% of the women partially utilized the health services while 11% adequately utilized the services. Partial utilization of health services was highest among women aged 25–34 years (69%) while adequate utilization was highest among women aged 35 years and above (12%). With respect to education, partial utilization was highest among women with primary education (76%) while adequate utilization was highest among women with secondary/higher education (20%). While most of the women who partially utilized health services were from South South (76%), most of those who adequately utilized the services were from Southeast (34%). Partial and adequate utilization of health services are highest among women from urban area (73% for partial and 18% for adequate) and those who are exposed to media (73% for partial and 15% for adequate).

**Table 1:** Utilization of health services by women of reproductive age in Nigeria

Variables	Maternal health services use					
	Attended Antenatal care		Delivered at health facility		Attended postnatal care	
	No N (%) 5,692 (26.1)	Yes N (%) 16,100 (73.9)	No N (%) 12,791(58.7)	Yes N (%) 9,001 (41.3)	No N (%) 17,153 (79.1)	Yes N (%) 4,531 (20.9)
Age						
15–24	1,573 (29.1)	3,826 (70.9)	3,525 (65.3)	1,874 (34.7)	4,409 (82.0)	966 (18.0)
25–34	2,471 (24.0)	7,816 (76.0)	5,806 (56.4)	4,481 (43.6)	7,985 (78.0)	2,251 (22.0)
35+	1,648 (27.0)	4,458 (73.0)	3,460 (56.7)	2,646 (43.3)	4,759 (78.4)	1,314 (21.6)
Education						
None	4,127 (43.3)	5,400 (56.7)	8,065 (84.7)	1,462 (15.3)	8,436 (88.6)	1,084 (11.4)
Primary	646 (18.9)	2,764 (81.1)	1,960 (57.5)	1,450 (42.5)	2,584 (76.1)	813 (23.9)
Secondary/higher	919 (10.4)	7,936 (89.6)	2,766 (31.2)	6,089 (68.8)	6,133 (70.0)	2,634 (30.0)
Marital status						
Never married	141 (23.3)	464 (76.7)	290 (47.9)	315 (52.1)	447 (74.4)	154 (25.6)
Ever married	5,551 (26.2)	15,636 (73.8)	12,501 (59.0)	8,686 (41.0)	16,706 (79.2)	4,377 (20.8)
Household wealth index						
Poorest	2,410 (48.0)	2,615 (52.0)	4,404 (87.6)	621 (12.4)	4,369 (87.0)	653 (13.0)
Poorer	1,633 (33.3)	3,272 (66.7)	3,662 (74.7)	1,243 (25.3)	4,144 (84.6)	753 (15.4)
Middle	893 (19.5)	3,693 (80.5)	2,545 (55.5)	2,041 (44.5)	3,542 (77.6)	1,024 (22.4)
Richer	500 (12.4)	3,525 (87.6)	1,536 (38.2)	2,489 (61.8)	2,874 (72.0)	1,120 (28.0)
Richest	256 (7.9)	2,995 (92.1)	644 (19.8)	2,607 (80.2)	2,224 (69.4)	981 (30.6)
Employment status						
Not working	2,457 (35.2)	4,520 (64.8)	4,879 (69.9)	2,099 (30.1)	5,916 (85.0)	1,044 (15.0)
Working	3,235 (21.8)	11,580 (78.2)	7,913 (53.4)	6,902 (46.6)	11,237 (76.3)	3,487 (23.7)
Preceding birth interval						
< 24 months	1,042 (28.6)	2,599 (71.4)	2,243 (61.6)	1,398 (38.4)	2,885 (79.6)	737 (20.4)
24 months and above	3,874 (26.9)	10,524 (73.1)	8,769 (60.9)	5,629 (39.1)	11,426 (79.7)	2,914 (20.3)
Residence						
Urban	1.007 (13.1)	6,703 (86.9)	2,955 (38.3)	4,755 (61.7)	5,427 (70.9)	2,224 (29.1)
Rural	4,685 (33.3)	9,397 (66.7)	9,836 (69.9)	4,246 (30.1)	11,726 (83.6)	2,307 (16.4)
Region						
North central	1,046 (27.0)	2,829 (73.0)	1,878 (48.5)	1,997 (51.5)	3,183 (82.2)	690 (17.8)
North east	1,283 (28.5)	3,223 (71.5)	3,337 (74.1)	1,169 (25.9)	3,767 (83.8)	726 (16.2)
North west	2,348 (37.2)	3,961 (62.8)	5,320 (84.3)	989 (15.7)	5,619 (89.1)	689 (10.9)
South east	118 (5.0)	2,247 (95.0)	478 (20.2)	1,887 (79.8)	1,306 (56.7)	999 (43.3)
South south	620 (28.5)	1,554 (71.5)	1,176 (54.1)	998 (45.9)	1,718 (79.3)	448 (20.7)
South west	277 (10.8)	2,286 (89.2)	602 (23.5)	1,961 (76.5)	1,560 (61.4)	979 (38.6)
Media exposure						
Not exposed	3,438 (40.4)	5,069 (59.6)	6,749 (79.3)	1,758 (20.7)	7,461 (87.9)	1,026 (12.1)
Exposed	2,254 (17.0)	11,031 (83.0)	6,042 (45.5)	7,243 (54.5)	9,692 (73.4)	3,505 (26.6)
Getting permission to use health service						
A problem	1,299 (48.8)	1,363 (51.2)	1,975 (74.2)	687 (25.8)	2,235 (84.3)	415 (15.7)
Not a problem	4,393 (23.0)	14,737 (77.0)	10,816 (56.5)	8,314 (43.5)	14,918 (78.4)	4,116 (21.6)
Getting money for treatment						
A problem	3,496 (32.4)	7,308 (67.6)	7,064 (65.4)	3,740 (34.6)	8,641 (80.4)	2,107 (19.6)
Not a problem	2,196 (20.0)	8,792 (80.0)	5,727 (52.1)	5,261 (47.9)	8,512 (77.8)	2,424 (22.2)
Distance to health facility						
A problem	2,575 (39.9)	3,879 (60.1)	4,559 (70.6)	1,895 (29.4)	5,250 (81.6)	1,181 (18.4)
Not a problem	3,117 (20.3)	12,221 (79.7)	8,232 (53.7)	7,106 (46.3)	11,903 (78.0)	3,350 (22.0)
Not willing to go to health facility alone						
A problem	1,522 (42.6)	2,052 (57.4)	2,600 (72.8)	974 (27.2)	2,887 (81.3)	665 (18.7)
Not a problem	4,170 (22.9)	14,048 (77.1)	10,191 (56.0)	8,027 (44.0)	14,266 (78.7)	3,866 (21.3)

**Table 2:** Health services utilization status by independent variables

Variable	Maternal health services use				
	Not utilized	Partially utilized	Adequately utilized	Total	P value
	N (%)	N (%)	N (%)	N (%)	
	4,677 (21.6)	14,731 (67.9)	2,276 (10.5)	21,684 (100.0)	
Age					
15–24	1,319 (24.5)	3,657 (68.0)	399 (7.4)	5,375 (100.0)	
25–34	2,001 (19.6)	7,070 (69.0)	1,165 (11.4)	10,236 (100.0)	
35+	1,357 (22.3)	4,004 (65.9)	712 (11.7)	6,073 (100.0)	< 0.001
Education					
None	3,706 (38.9)	5,623 (59.1)	191 (2.0)	9,520 (100.0)	
Primary	485 (14.3)	2,566 (75.5)	346 (10.2)	3,397 (100.0)	
Secondary/higher	486 (5.5)	6,542 (74.6)	1,739 (19.8)	8,767 (100.0)	< 0.001
Marital status					
Never married	95 (15.8)	429 (71.4)	77 (12.8)	601 (100.0)	
Ever married	4,582 (21.7)	14,302 (67.8)	2,199 (10.4)	21,083 (100.0)	< 0.001
Household wealth index					
Poorest	2,142 (42.7)	2,771 (55.2)	109 (2.1)	5,022 (100.0)	
Poorer	1,443 (29.5)	3,203 (65.4)	251 (5.1)	4,897 (100.0)	
Middle	684 (15.0)	3,368 (73.8)	514 (11.2)	4,566 (100.0)	
Richer	315 (7.9)	2,992 (74.9)	687 (17.2)	3,994 (100.0)	
Richest	93 (2.9)	2,397 (74.8)	715 (22.3)	3,205 (100.0)	< 0.001
Employment					
Not working	2,165 (31.1)	4,375 (62.9)	420 (6.0)	6,960 (100.0)	
Working	2,512 (17.1)	10,356 (70.3)	1,856 (12.6)	14,724 (100.0)	< 0.001
Preceding birth interval					
< 24 months	861 (23.8)	2,405 (66.4)	356 (9.8)	3,622 (100.0)	
24 months and above	3,217 (22.4)	9,728 (67.8)	1,395 (9.7)	14,340 (100.0)	0.202
Residence					
Urban	683 (8.9)	5,577 (72.9)	1,391 (18.2)	7,651 (100.0)	
Rural	3,994 (28.5)	9,154 (65.2)	885 (6.3)	14,033 (100.0)	< 0.001
Region					
North central	814 (21.0)	2,668 (68.9)	391 (10.1)	3,873 (100.0)	
North east	1,093 (24.3)	3,229 (71.9)	171 (3.8)	4,493 (100.0)	
North west	2,201 (34.9)	3,987 (63.2)	120 (1.9)	6,308 (100.0)	
South east	61 (2.7)	1,452 (63.0)	792 (34.3)	2,305 (100.0)	
South south	400 (18.5)	1,637 (75.6)	129 (5.9)	2,166 (100.0)	
South west	108 (4.3)	1,758 (69.2)	673 (26.5)	2,539 (100.0)	< 0.001
Media exposure					
Not exposed	3,072 (36.2)	5,118 (60.3)	297 (3.5)	8,487 (100.0)	
Exposed	1,605 (12.2)	9,613 (72.8)	1,979 (15.0)	13,197 (100.0)	< 0.001
Getting permission to use health service					
A problem	1,106 (41.7)	1,424 (53.7)	120 (4.5)	2,650 (100.0)	
Not a problem	3,571 (18.8)	13,307 (69.9)	2,156 (11.3)	19,034 (100.0)	< 0.001
Getting money for treatment					
A problem	2,923 (27.2)	6,894 (64.1)	931 (8.7)	10,748 (100.0)	
Not a problem	1,754 (16.0)	7,837 (71.7)	1,345 (12.3)	10,936 (100.0)	< 0.001
Distance to health facility					
A problem	2,160 (33.6)	3,825 (59.5)	446 (6.9)	6,431 (100.0)	
Not a problem	2,517 (16.5)	10,906 (71.5)	1,830 (12.0)	15,253 (100.0)	< 0.001
Not willing to go to health facility alone					
A problem	1,287 (36.2)	2,035 (57.3)	230 (6.5)	3,552 (100.0)	
Not a problem	3,390 (18.7)	12,696 (70.0)	2,046 (11.3)	17,342 (100.0)	< 0.001

### Correlates of partial and adequate utilization of health services

Table 3 shows results from the full adjusted multinomial logistic regression model. Results from the table indicate that the odds of partially and adequately utilizing health services increased respectively by a factor of 2.35 and 3.87 for women with primary education compared to women with no education. Also, the odds increased respectively by a factor of 4.21 and 10.39 for women with secondary/higher education. Ever married women were 46% and 64% respectively more likely to partially and adequately utilize health services compared to their counterparts who were never married. Working women were 48% and 72% respectively more likely to partially and adequately utilize health services compared to non-working women. With respect to household wealth, the odds of partially and adequately utilizing health services increased by a factor of 4.83 and 8.09 respectively for women from richest households compared to women from poorest households. Women living in urban area were 13% and 24% respectively more likely to partially and adequately utilize health services compared to their counterparts living in rural area. While the odds of partially utilizing health services increased by a factor of 2.84 and 2.06 respectively for women in Southeast and Southwest, the odds of adequately utilizing health services increased respectively by a factor of 7.89 and 3.94 for women in Southeast and Southwest compared to women in North Central. Women in Northeast also show positive inclination towards health services use as they are 93% more likely to partially utilize health services compared to women in North Central. However, for women in Northwest, the odds of adequately utilizing health services reduced by a factor of 0.29. Women who are exposed to media were 61% and 126% respectively more likely to partially and adequately utilize health services compared to women not exposed to media. Women who did not have problem in getting permission to use health services were 70% and 188% more likely to partially and adequately utilize health services compared to women who experienced problem getting permission. Women who did not have problem getting to health facility were 36% and 56% respectively more likely to partially and adequately utilize health services compared to women who experienced problem getting to health facility. The odds of partially utilizing health services increased by a factor of 1.16 for women who did not have problem visiting health facility unaccompanied compared to their counterparts who experienced problem visiting health facility alone.

**Table 3:** Results of multinomial logistic regression for maternal health services utilization in Nigeria

Variables	Maternal health services utilization	
	Partially utilized	Adequately utilized
Age	aOR (95% CI)	aOR (95% CI)
15–24	1	1
25–34	0.99 (0.91–1.09)	1.08 (0.92–1.27)
35+	0.86** (0.78–0.95)	1.04 (0.87–1.24)
Education		
No education	1	1
Primary	2.35*** (2.09–2.65)	3.87*** (3.09–4.85)
Secondary/higher	4.21*** (3.68–4.83)	10.39*** (8.32–12.98)
Marital status		
Never married	1	1
Ever married	1.46** (1.13–1.89)	1.64** (1.15–2.34)
Occupation		
Not working	1	1
Working	1.48*** (1.37–1.60)	1.72*** (1.49–1.98)
Household wealth index		
Poorest	1	1
Poorer	1.37*** (1.25–1.50)	1.81*** (1.41–2.33)
Middle	2.06*** (1.83–2.31)	3.13*** (2.43–4.04)
Richer	2.56*** (2.19-3.00)	4.06*** (3.08–5.36)
Richest	4.83*** (3.78–6.20)	8.09*** (5.74–11.42)
Residence		
Rural	1	1
Urban	1.13* (1.01–1.26)	1.24** (1.07–1.44)
Region		
North Central	1	1
North East	1.93*** (1.71–2.18)	1.09 (0.88–1.37)
North West	0.98 (0.88–1.09)	0.29*** (0.23–0.38)
South East	2.84*** (2.14–3.77)	7.89*** (5.79–10.74)
South South	0.44*** (0.38–0.52)	0.18*** (0.14–0.23)
South West	2.06*** (1.63–2.59)	3.94*** (3.02–5.13)
Media exposure		
Not exposed	1	1
Exposed	1.61*** (1.48–1.75)	2.26*** (1.92–2.66)
Getting permission to use health service		
A problem	1	1
Not a problem	1.70*** (1.52–1.90)	2.88*** (2.24–3.69)
Getting money for treatment		
A problem	1	1
Not a problem	1.07 (0.98–1.17)	1.02 (0.89–1.17)
Distance to health facility		
A problem	1	1
Not a problem	1.36*** (1.23–1.50)	1.56*** (1.31–1.86)
Not willing to go to health facility alone		
A problem	1	1
Not a problem	1.16* (1.04–1.30)	1.02 (0.83–1.27)

Level of significance at *p < 0.05, **p < 0.01, ***p < 0.001

## Discussion

Making use of nationally representative data set, this study examined the factors associated with maternal health services use during pregnancy, delivery and postnatal period. It is evident from the study that maternal health services utilization in Nigeria is still poor. While 74% of the women attended antenatal care, only 41% and 21% delivered at health facility and attended postnatal care respectively. This indicates that the proportions of women who attended antenatal care during pregnancy and had facility delivery thereafter diminished considerably. The proportions diminished further for postnatal care. The study also revealed that while 68% of the women partially utilized health services, only 11% adequately utilized the services.

Our study showed that women’s education plays an important role in health services use. Those who have primary and secondary/ higher education were more likely to partially and adequately utilize health services than those with no education. This dichotomy in health services use between educated and uneducated women has been emphasised in previous studies where it was stated that women with better education tend to have positive maternal health-seeking behaviour which enables them to attend antenatal care, deliver at health facility and report for postnatal care [30–37]. Education enhances the perspectives which an individual brings to different issues. Educated women appreciate the benefits of health services to themselves and their children.

Marital status showed a significant relationship with health services use. Ever married women were more likely to partially and adequately utilize health services compared to never married women. This has been reported in other studies and the reason provided in this regard is that married women tend to enjoy financial support from their husbands [38, 39]. Employment status is another factor which contributes significantly to health services utilization. Working women were more likely to partially and adequately utilize health services compared to non-working women. This may be due to financial autonomy advantage which working women have over their non-working counterparts [40, 41]. Use of health services come with some costs and women with limited financial resources may not access health service, particularly where the households too are financially constrained.

By extension, household wealth has been identified as one of the major determinants of women’s use of health services. Women from rich households utilized all the health services compared to their counterparts from poor households [21, 42–46]. The effects of poverty on women’s health services use may manifest in different ways. For instance, poor pregnant women may not attend antenatal care at all or may attend less than the required number of times. This, invariably, exposes such women and their foetus to poor care. As nutrition during pregnancy is an important component of pregnancy care, women from poor households may not have the means to consume nutritious food. This may later affect the pregnancy. Non-attendance of antenatal clinic is more often than not a precursor to non-facility delivery. This implies that poor women who do not attend antenatal clinic are most likely to deliver outside health facility. Women who neither attended antenatal clinic nor delivered at health facility are likely to not attach importance to postnatal care.

The study further showed that urban women were more likely to partially and adequately utilize health services than rural women. Women in the urban area have some advantage over their rural counterparts in that the number of health facility in the former is more than the latter. At the same time, transport system in the urban area makes it easier for urban dwellers to reach health facility unlike rural dwellers. This has also been corroborated by previous studies in which it was opined that women in urban areas are better placed in terms of information, knowledge and access to maternal health services as against rural women [47–51].

Use of health services varies geographically in the country as women in Southwest and Southeast have higher tendency of partially and adequately utilizing all the health services compared to women from North Central and other regions. One distinguishing characteristic between the two regions and others is high proportions of educated women [15]. This, among other factors, could have contributed immensely to use of health services in the two regions. For women in Northeast who partially utilized health services, this may be attributed to other factors such as exposure to information on maternal health care and special awareness programme on health services use at community level.

Information obtained through media tends to increase use of health services as women who are exposed to media were more likely to partially and adequately utilize health services. This may be linked to recent developments in the country where awareness programmes on maternal and child health are regularly presented on electronic media. Such programmes offer listeners and viewers the opportunity to obtain vital information which ordinarily would have eluded them. Analysis from earlier studies also emphasized that mass media could have a significant impact on the reduction of maternal mortality and morbidity through the broadcast of maternal health care related information to women of reproductive age [52–55].

Issues relating to seeking permission also came up in the study. Women who did not encounter problems in getting permission from their spouses to visit health facility partially and adequately utilized health services more than women who experienced problems in this regard. Cultural practices and religious dictates in some parts of the country require women to seek permission of their husbands when going out of the households. In some studies, it was stated that many women still have to obtain permission before utilizing health service and that a delay in the permission being granted may have negative effects on the use of health services by such women [56].

This also relates to unwillingness to go to health facility alone. Partial and adequate utilization of health services was poor among women who complained that they were prevented from utilizing health services because they did not have somebody that would accompany them to the health facility. This has been identified in previous studies where it was re-iterated that in some culture, female relatives play important roles such as teaching women traditions, escorting women to health facility and providing emotional support [57]. In a situation where there are no relatives to provide such roles, health services utilization may be adversely affected.

Distance to health facility influences the use of health services because women who never experienced difficulty in getting to health facility partially and adequately utilized health services compared to women who experienced problem getting to health facility. Some women reside in remote areas with bad roads and poor transport system. Such women would not be able to access health services as regularly as they may desire [58–61].

### Policy implications

Since sustainable development goal 3 focuses on health and well-being, reduction of maternal mortality is one of the ways of achieving this goal. This is why emphasis has been placed on reduction of maternal mortality ratio to less than 70 per 100,000 live births by 2030. This in itself requires adequate utilization of health services by women. The 11% adequate utilization of health services by women indicates that much still needs to be done in Nigeria to be on track of achieving the goal.

To put the country on track, some measures need to be adopted. In the first instance, the existing programmes aimed at improving health services utilization should be evaluated and necessary adjustments should be made particularly in areas where progress has been stalled. Efforts should be geared towards revising the free education policy in order to accommodate items that would promote increase in enrolment especially among females. For instance, the policy should be extended to provision of text books free of costs to students so that children from poor households would be catered for. This, however, would require adjustments in budget as the present budgetary allocations for education at federal and state levels are grossly inadequate.

Addressing the problem of poverty at household level should be a major concern. Attempts should be made to create enabling environment for members of households who furnish the idea of self-employment and funds should be made available for those who wish to engage in small and medium scale enterprises. Those involved in agricultural activities should be supported through provision of interest-free loans for the procurement of necessary equipment. Based on the effects of media exposure on health services utilization, awareness programme in respect of maternal and child health being sponsored in the media should be strengthened and sustained.

More so, regional disparities in health services utilization should be addressed by state governments. Some states in the Southwest of the country earmarked special funds for maternal and child health programme to the extent of building special centre for such purpose. This has contributed immensely to improvement in health services utilization in the states. Other states should embark on similar project to improve maternal and child health. At federal and state levels, there is a need to invest in provision of more health facilities in rural and remote areas. In addition, construction of access roads for easy mobility to such facility should be given adequate attention.

Issues relating to seeking permission to visit health facility and being accompanied to health facility should be addressed at the community level. Health awareness seminar that would involve community and religious leaders, health workers and married men and women should be organised on regular basis. At such gathering, emphasis should be placed on the role of men in maternal and child health.

It is important to also state that these suggestions should be brought to the attention of policymakers for proper implementation. One of the ways of achieving this is by involving members of State and Federal Houses of Assembly from their constituencies especially on those suggestions that require government implementation. Such legislators should be enjoined to present the issues at the floor of the House and discuss them at meetings of Committees performing oversight functions in respect of different ministries. The legislators may also use their goodwill to interact with members of the executive governments at state and federal levels. In addition, it would be worthwhile to prepare a policy brief summarising the findings from this study, including policy implications, which should reach the head of concerned ministries for onward consideration.

### Study strengths and weaknesses

Findings in this study are based on secondary data obtained from survey that is conducted on regular basis. This provides consistent access to information relating to maternal health indicators. As a result of wide coverage of the survey, the data are nationally representative which makes it possible to generalise the findings. Most previous studies treated maternal health services use by considering antenatal care, delivery or postnatal care separately. But our study adopted a different approach by combining the three maternal health services and presenting such as the outcome variable using a single analytical framework. This contributed to the robust findings in the study. However, since the survey is based on interviews in which questions on past events are asked, recall error may occur. Parity is an important factor influencing maternal health services use which the study did not include. At the same time, our study did not consider interaction effects among variables which could have provided more information on the relationship between the outcome and independent variables. The cross-sectional nature of the survey prevented us from assessing the drop-outs of women at each stage of maternity care and the factors influencing this in a longitudinal manner.

## Conclusions

This study has revealed the factors associated with partial and adequate utilization of health services among women in Nigeria. Such factors include education, household wealth, marital status, employment status, residence, region, media exposure, getting permission to use health service, unwillingness to visit health facility alone and distance to health facility. Improvement in maternal health services utilization requires adequate attention to be paid to interventions that would address the challenges posed by these factors.

## Electronic Supplementary Material

Below is the link to the electronic supplementary material.

**Additional file 1:** Review history.

## Data Availability

Data used in this study were obtained from the DHS Program and available at: http://dhsprogram.com/data/dataset/Nigeria_Standard-DHS_2013.cfm?flag=0. The survey was approved by Institutional Review Board (IRB) of ICF Macro in the United States and the National Ethics Committee in the Federal Ministry of Health of Nigeria.

## List of Abbreviations

aOR: Adjusted Odds Ratio
CI: Confidence Interval
OR: Odds Ratio
WHO: World Health Organization

## Declarations

### Review history

The review history is available as Additional file 1.
