# Supplementary material for: Determinants of partial and adequate maternal health services utilization in Nigeria: analysis of cross-sectional survey
Source: BMC Pregnancy Childbirth. 2023 Jun 20;23:457. doi: 10.1186/s12884-023-05712-4 (PMC10283328; doi:10.1186/s12884-023-05712-4)
Supplement: Supplementary file 1 — Additional file 1: Review history. [file 12884_2023_5712_MOESM1_ESM.docx]

# Version 1

## Reviewer 1:

In general, this is a clear, concise, and well-written manuscript. The introduction is relevant and theory-based. Sufficient information about the previous study findings is presented for readers to follow the present study rationale and procedures. The methods are generally appropriate, although clarification of a few details and provision of a rationale for the use of this particular method of measuring the variables of maternal health services utilization. Specific comments follow.

Introduction

•	The authors provided detailed data on maternal health services utilization in the sub-Saharan Africa region. It is good to start the paragraph (Paragraph 2) with global data and/or other regions e.g Southeast Asian region – good to read any comparison of various data from other regions.

•	Perhaps the authors should be able to provide any supporting references in this part, as to why this study merely focused on three main maternal health services mentioned in the introduction section.

Methods

•	Need clarification: did the authors use the secondary data of the previous survey (Nigeria Demographic and Health Survey (NDHS) 2018? Or did the authors conduct a new study using available sampling which has been used for the previous surveys? From my understanding, this study was conducted using the dataset.

•	Concerning the use of the research instrument (questionnaire), did the authors use a standard questionnaire or develop a new instrument? If it was a new one, it is good to write down how the instrument was generated and pre-tested.

•	Any ethical clearance required? Kindly provide this in some parts of the section.

•	How do the authors control bias resulting from the interviews? As the authors mentioned in the weaknesses section recall error may occur during the interviews.

Policy implications

•	“Achieving the sustainable development goal 3 which emphasises the reduction of maternal mortality ratio to less than 70 per 100,000 live births by 2030 requires adequate utilization of health services by women.” Goal 3 of SDGs is not only focused on maternal health issues, it is about health and well-being. Yes, MMR is one of the indicators of this goal. Kindly revise this sentence in order to be more concise.

•	Apart from focusing on the policy, also good to write about how policymakers should be aware of this important issue.

## Reviewer 2:

Study Title: Determinants of partial and adequate maternal health services utilization in Nigeria: Analysis of a cross sectional survey

Summary

This paper assesses the factors associated with maternal health utilization using the 2018 Demographic and Health survey (NDHS). An analysis was done with data collected from 21,792 women aged 15-49 who gave birth within five years of the survey. The study focused on multiple outcomes related to antenatal, delivery and postnatal care and applying them to a number of independent variables such as age, educational status etc.

Strengths of the study

o	Good statistical analysis with clear outcome variables and independent variables outlined

o	Results section laid out clearly with these also detailed clearly in three tables

o	Good discussion section relating to findings and relating them to previously published papers in this field

Weaknesses/Major Issues

o	The methods section describes the Nigeria Demographic Health Survey (NDHS ) in great detail but this methodology was not designed or conducted by the authors. The authors should have focused on how they went about their analysis in the methods section.

o	It is not very clear how the definitions of partial and adequate utilisation of services were defined and what supported this definition.

o	The NDHS survey was conducted in 2015/16 and published in 2018 and we are in 2022/23. This survey may be considered a bit old.

o	There are other surveys which are done more frequently and more recent e.g. Multiple Indicators Cluster Survey (MICS) that could also be considered.

o	The study does not offer any new knowledge

Minor Issues

o	The analysis referencing regional differences should also speak to North East, North West regions specifically as they exhibit major challenges of these factors that effect utilization of maternal health services

o	Spell check required and repetition of words in some places. Requires editing if eventually accepted.

Recommendations

The manuscript could be revised by responding to the weaknesses/major issues. However, my personal view is that the content of the manuscript does not add any new knowledge to the issues being assessed.

## Authors’ response:

The Editorial Team,

BMC Women’s Health,

Re: Determinants of partial and adequate maternal health services utilization in Nigeria:

Analysis of cross-sectional survey

We are pleased to submit a revised manuscript referenced above according to the reviewers’

comments. We have made every effort to revise our manuscript based on the reviewers’

suggestions and recommendations, which we believe made our paper stronger and better.

In the following pages, we provide a point-by-point response to the reviewers’ comments.

We have put the reviewers’ comments in bold with our responses below each comment.

We thank the reviewers for the pertinent comments, which helped to improve the quality of

the manuscript. Generally, your remarks were very useful and stimulating. Our responses to

the points are hereby presented below.

We would like to thank you in advance for your consideration.

Yours faithfully,

Dr. Sulaimon T. Adedokun

For Authors

REVIEWERS’ COMMENTS AND AUTHORS’ RESPONSES

Reviewer 1:

In general, this is a clear, concise, and well-written manuscript. The introduction is

relevant and theory-based. Sufficient information about the previous study findings is

presented for readers to follow the present study rationale and procedures. The

methods are generally appropriate, although clarification of a few details and provision

of a rationale for the use of this particular method of measuring the variables of

maternal health services utilization.

Response: Thank you for these encouraging comments.

Specific comments follow

Introduction

 The authors provided detailed data on maternal health services utilization in the

sub-Saharan African region. It is good to start the paragraph (Paragraph 2) with

global data and/or other regions e.g Southeast Asian region- good to read any

comparison of various data from other regions.

Response: Thank you. We have corrected this and included some global data and some

regions in the paragraph

 Perhaps the authors should be able to provide any supporting references in this

part as to why this study merely focused on three main maternal services

mentioned in the introduction section.

Response: We have provided reference supporting our choice of the three main maternal

services in the introduction section

Methods

 Need clarification: did the authors use the secondary data of the previous survey

(Nigeria Demographic and Health Survey (NDHS) 2018? Or did the authors

conduct a new study using available sampling which has been used for the

previous surveys? From my understanding, this study was conducted using the

dataset.

Response: Yes, we used the Nigeria Demographic and Health Survey 2018 dataset which is a

secondary dataset. We have included a statement indicating that we have used a secondary

dataset. Also, we have removed some items in the sampling procedure section so that it

would not appear as if we collected the data.

 Concerning the use of the research instrument (questionnaire), did the authors

use a standard questionnaire or develop a new instrument? If it was a new one, it

is good to write down how the instrument was generated and pre-tested.

Response: Since we used secondary data, we did not develop any instrument. We only

described the questionnaire used in the NDHS which is a standardized questionnaire. We

have also included it in method section that it is a standardized questionnaire.

 Any ethical clearance required? Kindly provide this in some parts of the section.

Response: The study did not require any ethical clearance as we have used secondary data.

However, information about ethical clearance has been added in the Methods section and

Declarations section.

 How do the authors control bias resulting from the interviews? As the authors

mentioned in the weaknesses section recall error may occur during the

interviews.

Response: We could not control bias that occurred on the part of the respondents particularly

recall error and that is why we stated this as one of the weaknesses. We controlled for errors

that occurred through over-reporting and under-reporting in the survey using the weighting

factor as stated in the methods section.

Policy implications

 “Achieving the sustainable development goal 3 which emphasises the reduction

of maternal mortality ratio to less than 70 per 100,000 live births by 2030

requires adequate utilization of health services by women.” Goal 3 of SDGs is

not only focused on maternal health issues, it is about health and well-being. Yes,

MMR is one of the indicators of this goal. Kindly revise this sentence in order to

be more concise.

Response: The sentence has been revised as suggested.

 Apart from focusing on the policy, also good to write about how policymakers

should be aware of this important issue.

Response: We have included points on how this issue would reach policymakers.

Reviewer 2

Study Title: Determinants of partial and adequate maternal health services utilization

in Nigeria: Analysis of a cross sectional survey

Summary

This paper assesses the factors associated with maternal health utilization using the

2018 Demographic and Health survey (NDHS). Analysis was done with data collected

from 21,792 women aged 15-49 who gave birth within five years of the survey. The

study focused on multiple outcomes related to antenatal, delivery and postnatal care

and applying them to a number of independent variables such as age, educational status

etc.

Response: Thank you for these observations

Strengths of the study

o Good statistical analysis with clear outcome variables and independent variables

outlined

Response: Thank you

o Results section laid out clearly with these also detailed clearly in three tables

Response: Thank you

o Good discussion section relating to findings and relating them to previously

published papers in this field.

Response: Thank you

Weaknesses/Major Issues

o The methods section describes the Nigeria Demographic and Health Survey

(NDHS) in great detail but this methodology was not designed or conducted by

the authors. The authors should have focused on how they went about their

analysis in the methods section.

Response: We have removed some information in the methods section particularly under the

study design and sampling procedure section.

o It is not very clear how the definitions of partial and adequate utilisation of were

defined and what supported this definition.

Response: We have clearly explained how the outcome variable was defined and how the

three categories of not utilized, partially utilized and adequately utilized were arrived at. We

have also included evidence supporting such definition.

o The NDHS survey was conducted in 2015/2016 and published in 2018 and we are

in 2022/23. This survey may be considered a bit old.

Response: Thank you. The NDHS survey was not conducted in 2015/16. The survey started

in August 14, 2018 and lasted till December 29, 2018. It was in 2019 that the data were

released. This information is contained in the NDHS final report. As at the time of preparing

this manuscript, the survey was the latest NDHS for Nigeria. We also intend doing a followup study looking at the trends and pattern of maternal health services utilization by making

use of three most recent NDHS datasets.

o There are other surveys which are done more frequently and more recent e.g.

Multiple Indicators Cluster Survey (MICS) that could also be considered.

Response: Thank you. We acknowledge the fact that there are other surveys but each survey

has its peculiarity in terms of focus and variables. NDHS is a survey with the most variables

and focus which suit the purpose of our study.

o The study does not offer any new knowledge.

Response: Our study adopted an approach which most previous studies did not use and as

such provided robust findings on the factors associated with maternal health services use in

Nigeria. We highlighted this in the introduction section and the strengths and weaknesses

section.

Minor Issues

o The analysis referencing regional differences should also speak to North East,

North West regions specifically as they exhibit major challenges of these factors

that affect utilization of maternal health services.

Response: We have included information on Northeast and Northwest in the results and

discussion sections.

o Spell check required and repetition of words in some places. Requires editing if

eventually accepted.

Response: We have effected changes where necessary.

Recommendations

The manuscript could be revised by responding to the weaknesses/major issues.

However, my personal view is that the content of the manuscript does not add any new

knowledge to the issues being assessed.

Response: We have responded to the weaknesses/major issues according to our ability. In our

study, we adopted a different approach to the study of maternal health services use which has

contributed immensely to a better understanding of the factors influencing maternal health

services utilization in Nigeria.

# Version 2

## Reviewer 1:

Discussion and policy implication sections consist only one long paragraph. Could the authors please break down into several parts according to the main topic of each paragraph.

## Reviewer 2:

The revised manuscript has taken on board the comments and suggestions from the initial review.

## Authors’ response:

The Editorial Team,

BMC Women’s Health,

Re: Determinants of partial and adequate maternal health services utilization in Nigeria:

Analysis of cross-sectional survey

We are pleased to submit a revised manuscript referenced above according to the reviewers’

comments. We have made every effort to revise our manuscript based on the reviewers’

suggestions and recommendations, which we believe made our paper stronger and better.

In the following pages, we provide a point-by-point response to the reviewers’ comments.

We have put the reviewers’ comments in bold with our responses below each comment.

We thank the reviewers for the pertinent comments, which helped to improve the quality of

the manuscript. Generally, your remarks were very useful and stimulating. Our responses to

the points are hereby presented below.

We would like to thank you in advance for your consideration.

Yours faithfully,

Dr. Sulaimon T. Adedokun

For Authors

REVIEWERS’ COMMENTS AND AUTHORS’ RESPONSES

Reviewer 2:

The revised manuscript has taken on board the comments and suggestions from the

initial review.

Response: Thank you.

Reviewer 1

Discussion and policy implication sections consist only one long paragraph. Could the

authors please break down into several parts according to the main topic of each

paragraph.

Response: Thank you. We have broken the discussion and policy implication sections down

into several paragraphs as suggested.

# Version 3

## Reviewer 1:

All good, best of luck for the authors

## Reviewer 3:

Title - Determinants of partial and adequate maternal health services utilization in Nigeria: Analysis of cross-sectional survey

Title of manuscript – Appropriate

Abstract – Well-structured with appropriate content

Introduction – Statement of problem, justification for the study and the study objectives well presented

Methods - Study designed well described

•	Study population, clear description of secondary data used.

•	Data Analysis appropriate

Result – Well presented in tables. Analysis correctly done

Discussion – Study findings well described. The strength and limitations stated in the discussion are valid.

First paragraph of the discussion - Compare the utilization level with what was reported in others studies in Nigeria and other countries.

Conclusion – Appropriate to the study findings

References – Recent and adequate. Mention authors first in reference Nos. 1,2, 4-7, 12, 25 even if they are organisations
